# Overcoming barriers, driving progress: Clinical science at IAS 2025

**DOI:** 10.1002/jia2.70088

**Published:** 2026-03-23

**Authors:** Ezequiel Cordova, Simiso Sokhela, Jasmini Alagaratnam, Juan Ambrosioni

**Affiliations:** ^1^ Infectious Diseases Unit Hospital Cosme Argerich Buenos Aires Argentina; ^2^ Clinical Research Unit Fundación IDEAA Buenos Aires Argentina; ^3^ Ezintsha University of the Witwatersrand Johannesburg South Africa; ^4^ Department of HIV Medicine & Sexual Health Chelsea & Westminster Hospital NHS Foundation Trust London UK; ^5^ Section of Virology Department of Infectious Disease Imperial College London London UK; ^6^ HIV Unit Infectious Disease Service Hospital Clinic‐IDIBAPS University of Barcelona Barcelona Spain; ^7^ CIBERINFEC ISCIII Madrid Spain

**Keywords:** AIDS, ART, comorbidities, HIV, IAS, IAS 2025, Kigali, mpox, Rwanda, special populations, TB, tuberculosis

## Abstract

**Introduction:**

The 13th IAS Conference on HIV Science, held in Kigali, Rwanda (13–17 July 2025), highlighted key advances in clinical research. Presentations focused on sustaining HIV treatment and prevention amid financial constraints, innovations in long‐acting oral antiretrovirals, and the management of comorbidities and co‐infections, particularly tuberculosis (TB) and mpox.

**Discussion:**

Significant progress was reported on long‐acting oral antiretrovirals, including a weekly treatment regimen and a promising monthly option for pre‐exposure prophylaxis. These strategies could expand the current antiretroviral therapy (ART) portfolio to better meet individual needs. Additionally, the use of currently available long‐acting regimens in non‐suppressed individuals warrants further exploration, supported by growing evidence of their potential in this clinical context. Intermittent ART, previously studied as a means to reduce toxicity, is now gaining attention as a potential cost‐saving strategy. However, more research is needed to define its role across diverse settings. While data from high‐income countries is encouraging, results have been less favourable in resource‐limited settings and among key populations such as adolescents. Two‐drug and injectable regimens, increasingly used and supported by international guidelines in high‐income settings, are now being explored in resource‐limited contexts and incorporated into clinical guidelines—narrowing the gap between recommendations for high‐ and low‐income regions. As the ART portfolio evolves towards regimens without tenofovir, hepatitis B reactivation emerged as a key topic at the conference. Management of TB, a long‐standing clinical challenge, was also addressed in Kigali with trial data supporting early empiric TB treatment and the safety of same‐day ART initiation in selected clinical scenarios. The UNITY trial on tecovirimat for mpox treatment showed no significant clinical benefit, underscoring the need to revise current management guidelines. Research on comorbidities examined ART‐associated weight gain, showing that switching ART once obesity is established has a limited impact on weight outcomes. Studies in paediatric populations highlighted predictors of treatment failure and the benefits of dolutegravir‐based regimens.

**Conclusions:**

In the face of growing economic pressures, innovation in HIV treatment and prevention remains essential. The conference emphasized the importance of sustainable public health strategies and individualized care approaches to ensure continued progress in the global HIV response.

## 1 INTRODUCTION

According to UNAIDS estimates, 40.8 million people were living with HIV at the end of 2024. Despite progress in treatment and prevention, 9.2 million of these individuals were not receiving antiretroviral therapy (ART), and an estimated 1.3 million people newly acquired HIV, half of them in sub‐Saharan Africa [1]. These figures may worsen significantly due to recent political developments and funding cuts, most notably the discontinuation of support from the United States President's Emergency Plan for AIDS Relief (PEPFAR), which threatens decades of progress in the field [2].

Against this backdrop, the 13th IAS Conference on HIV Science was held in Kigali, Rwanda, from 13 July to 18 July, 2025. The conference presented significant new clinical data, with a strong focus on addressing current funding challenges, while maintaining a constructive and optimistic tone. More than 3000 participants attended in person, with over 500 joining virtually. In this article, we provide a critical assessment of the most relevant original clinical science presented at the conference and discuss its implications within the current context, as well as its potential to inform future improvements in HIV medicine. This review focuses exclusively on studies selected for oral presentations during the conference; within these, greater emphasis is placed on those considered most relevant by the Track B rapporteurs. Studies related to HIV prevention, including pre‐exposure prophylaxis (PrEP), are not included, as they were covered within a different conference track.

## 2 DISCUSSION

The key findings of the studies discussed below are summarized in Table 1.

**Table 1:** Main findings and critical assessment of selected clinical science presentations

Title of the presentation Antiretrovirals	First author/Reference	Session	Main findings	Critical assessment
Long‐acting cabotegravir and rilpivirine in adults with suboptimal HIV control in sub‐Saharan Africa: the IMPALA trial 48‐week results	Cresswell, F/3 Bahemuka, U/7	OAS0105LB OAB0202	The IMPALA phase trial demonstrated that long‐acting injectable CAB+RPV was non‐inferior to daily oral ART in maintaining viral suppression at 48 weeks among adults in sub‐Saharan Africa with prior suboptimal HIV‐1 control (HIV‐1 RNA >1000 copies/ml despite being on ART adherence challenges. While virological failure was rare, CAB+RPV‐LA significantly reduced the proportion of participants with viral loads >1000 copies/ml, supporting its potential for lowering transmission risk. With high participant preference and a favourable safety profile, CAB+RPV‐LA presents a promising alternative for improving ART adherence and outcomes in this setting. There was only one incident case of HBV in the CAB+RPV LA arm.	The study targets a high‐need population, filling a key gap in the evidence base for long‐acting injectable ART, and the design was appropriate for evaluating effectiveness in a pragmatic setting and enhances generalizability. Limitations include the open‐label nature of the study, which may have introduced bias. With few confirmed virological failure cases, conclusions about resistance risk and long‐term durability are limited. Longer follow‐up data are needed to evaluate durability of suppression, cumulative resistance risk and implementation challenges.
Real‐world effectiveness of CAB+RPV LA in individuals with HIV viraemia at therapy initiation	Hsu, R/4	OAB0104	Outcomes from the OPERA cohort of 368 viraemic individuals initiating CAB+RPV LA. Most participants were on INSTIs at the time of CAB+RPV LA initiation: 88% achieved suppression, and 1% experienced virologic failure.	Real‐world data support expanding CAB+RPV LA use in selected viraemic individuals with adherence or tolerability issues to oral regimens. Careful selection based on treatment history is essential.
First experience of dual antiretroviral maintenance regimen in West and Central Africa compared with continuing three‐drug current antiretroviral regimens: week 96 results from the MODERATO trial (ANRS‐MIE 12372)	Fadiga, F/8	OAB0103	In this RCT, dual therapy with DTG/3TC or ATV/r‐3TC was non‐inferior to triple therapy at week 96 with comparable safety and CD4 outcomes.	Dual therapy in sub‐Saharan African countries showed sustained virological control, reinforcing its indication as a switch option for suppressed people with HIV in the recently updated 2025 WHO guidelines.
A double‐blind, active‐controlled, phase 2b study to evaluate the efficacy and safety of ulonivirine in combination with islatravir in virologically suppressed adults living with HIV‐1	Molina, JM/12	OAB0102	Once‐weekly ISL+ULO maintained virological suppression through Week 24 in all participants, but was associated with reversible CD4+ and lymphocyte count declines, leading to early study discontinuation	Weekly oral regimens are an attractive dosing strategy. Despite the antiviral efficacy of ISL+ULO, safety concerns related to lymphocyte toxicity arose. A new formulation with a lower ISL weekly dose is currently under investigation.
Safety and pharmacokinetics of MK‐8527 oral once‐monthly: a phase 2 study in adults at low risk of HIV‐1 exposure	Mayer, K/14	OAS0106LB	MK‐8527, a once‐monthly oral PrEP, was well tolerated with a safety profile comparable to placebo and no significant impact on CD4 or lymphocyte counts. The 6 and 12 mg doses achieved protective drug levels, and no HIV acquisitions occurred during the study. These results support further development of MK‐8527 as a convenient, long‐acting oral PrEP option, with phase 3 trials planned for higher‐risk populations.	The study's strengths include its randomized, double‐blind design and comprehensive pharmacokinetic analysis, providing robust safety and efficacy signals for MK‐8527. Further phase 3 trials in people with higher risk are needed to confirm efficacy and acceptability in diverse, real‐world settings. As a once‐monthly oral agent, MK‐8527 represents a particularly attractive potential model for future oral long‐acting strategies.
Systematic review and meta‐analysis of the efficacy of intermittent antiretroviral therapy dosing: a crisis response to the sudden cuts in USAID and PEPFAR funding	Hill, A/18	OAB0106LB	Intermittent ART (3–5 days/week) showed equivalent 48‐week virologic efficacy to daily ART in selected, adherent, virologically suppressed individuals.	This meta‐analysis of intermittent ART provides evidence to support dosing flexibility during ART supply disruptions. Generalization of these results in real‐world settings, especially in low‐ and middle‐income countries, where historical virological data is more limited, cannot be warranted.
Short‐cycle antiretroviral therapy with weekends off is inferior to continuous ART in adolescents living with HIV receiving tenofovir disoproxil fumarate/lamivudine/dolutegravir (TLD) in sub‐Saharan Africa: BREATHER Plus 96‐week results	Kekitiinwa, A/19	OAS0104LB	BREATHER Plus study showed that short‐cycle therapy (5 days on/2 days off TLD) was inferior to continuous ART in virologically suppressed adolescents in sub‐Saharan Africa, with higher rates of viral rebound (10% vs. 5%, *p*=0.034). Despite similar adherence and safety, infrequent viral load monitoring likely contributed to poorer outcomes. Short‐cycle therapy cannot be recommended in this setting under current monitoring practices.	Short‐cycle intermittent TLD therapy in adolescents under standard viral load monitoring in this setting cannot be recommended. Further information on adherence measurement and age subgroup analysis is anticipated.
**Co‐infections and comorbidities**				
Tecovirimat for mpox—primary clinical outcome results from the randomized part of the Unity trial	Grinsztejn, B/20	OAM01	Phase III, multi‐country, double‐blind, placebo‐controlled superiority trial evaluating tecovirimat for mpox (Clade II). Primary endpoint was complete resolution of lesions. Tecovirimat was well tolerated but did not significantly accelerate lesion resolution.	These results corresponded to the randomized part of the study. People with more severe disease or at risk of complications were not randomized and all received tecovirimat. However, this is the third negative trial with tecovirimat for mpox treatment. Scientific societies should consider carefully their recommendations.
A two‐by‐two factorial randomized trial of immediate and/or sepsis‐specific dose anti‐tuberculosis therapy to improve mortality for adults with sepsis and HIV in East Africa	Heysell, S/22	OAB0502	Among PWH with sepsis and confirmed TB, immediate conventional‐dose or diagnosis‐dependent high‐dose anti‐TB therapy reduced 28‐day mortality versus standard care.	Interpretation limited by setting. Results support empiric TB therapy in high‐burden contexts or in PWH with high pre‐test probability, but are not generalizable to low‐incidence settings or populations with broader sepsis aetiologies.
Same‐day antiretroviral therapy initiation in people with HIV and presumptive tuberculosis: a randomized, non‐inferiority trial in Lesotho and Malawi (SaDAPT)	Gerber, F/23	OAB0503	Same‐day ART was non‐inferior to deferred ART in PWH with presumptive TB, with similar viral suppression, safety and retention outcomes at 26 weeks	Findings support same‐day ART initiation in people with presumptive TB, reinforcing current WHO guidelines. This is particularly relevant in settings where timely TB diagnosis is not feasible, helping to avoid unnecessary delays in treatment initiation.
Predictors of weight gain among people with HIV over 3‐year period	Elion, R/25	OAB0204	In this cohort, significant weight gain (≥10% with a BMI category shift) among virally suppressed people with HIV in the United States was primarily associated with socio‐demographic and clinical factors rather than antiretroviral therapy type or exposure. This suggests that weight gain in this population mirrors broader societal patterns and supports the need for targeted, non‐ART‐specific obesity interventions.	This large, well‐powered observational study provides valuable real‐world insights, particularly by using multivariate and machine learning analyses to robustly assess predictors of clinically significant weight gain. However, as with all retrospective designs, unmeasured confounding and reliance on patient record data may limit causal interpretations and underestimate the role of specific ART regimens or adherence patterns.
A5391: a randomized multicentre three‐arm controlled trial for people with obesity on integrase inhibitors and tenofovir alafenamide switching to doravirine, with or without tenofovir disoproxil fumarate (The DO‐IT Trial)	Koethe, J/26	OAB0206LB	The ACTG A5391 “DO‐IT” trial found that switching from INSTI+TAF/FTC to doravirine‐based regimens did not result in significant weight loss at 48 weeks among people with HIV and obesity. Weight changes were modest across all arms, with no statistically or clinically meaningful differences, highlighting that switching ART alone may not be sufficient to address weight gain in this population.	This well‐designed randomized trial provides valuable evidence from a diverse cohort, including a high proportion of women and Black participants, strengthening its relevance to populations disproportionately affected by HIV and weight‐related comorbidities. However, the study's relatively small sample size and short follow‐up period may limit its ability to detect more subtle or long‐term effects of switching ART on weight outcomes.
**Special populations**				
Predictors of treatment failure in children living with HIV starting first‐line antiretroviral therapy in the ODYSSEY trial.	Wyncoll J/27	OAB0301	The Odyssey trial evaluated predictors of virological failure in paediatric populations. ART other than DTG‐based regimens, younger age, low BMI for age and lower CD4% at ART initiation as key predictors of treatment failure.	The study highlights the importance of second‐generation INSTI‐based regimens in children, making them the preferred option for people with HIV in all stages of life, from newborns to older people with HIV.
Pregnancy and neonatal outcomes following prenatal exposure to cabotegravir: data from The Antiretroviral Pregnancy Registry (APR)	Vannappagari, V/28	OAB0402	Antiretroviral Pregnancy Registry data from 42 pregnancies with CAB exposure showed no safety signals.	Pregnancy outcomes in women exposed to CAB appear to show no safety signals, which is important given the expanding use of CAB‐LA for HIV treatment and prevention among women. However, the limited sample size precludes definitive conclusions, and continued monitoring through pregnancy registries remains essential to inform clinical guidance.

**Abbreviations**: 3TC, lamivudine; ART, antiretroviral therapy; ATV/r, atazanavir/ritonavir; BMI, body mass index; CAB, cabotegravir; DTG, dolutegravir; FTC, emtricitabine; INSTI, integrase strand transfer inhibitor; ISL, islatravir; LA, long‐acting; PrEP, pre‐exposure prophylaxis; PWH, people with HIV; RCT, randomized controlled trial; RPV, rilpivirine; TAF, tenofovir alafenamide; TB, tuberculosis; TDF, tenofovir disoproxil fumarate; ULO, ulonivirine.

### 2.1 Antiretrovirals

Several studies presented at the conference addressed different ART strategies, ranging from long‐acting (LA) injectable and oral regimens to two‐drug maintenance approaches, novel agents and intermittent dosing models, reflecting ongoing efforts to optimize treatment effectiveness, feasibility and sustainability across diverse settings.

The IMPALA trial further explored LA cabotegravir and rilpivirine (CAB+RPV) in adults in sub‐Saharan Africa with suboptimal HIV‐1 control (HIV‐1 RNA >1000 copies/ml despite being on ART), the majority of whom had prior exposure to non‐nucleoside reverse transcriptase inhibitor (NNRTI)‐based regimens (78.3%) [3]. Among 540 participants, after a period on oral therapy, CAB+RPV LA was non‐inferior to daily dolutegravir (DTG)‐based ART for viral suppression at 48 weeks. Confirmed virological failure was low, and all affected individuals resuppressed on oral ART, which was tenofovir, lamivudine and dolutegravir (TLD) in most cases, a very relevant aspect for CAB+RPV LA use in limited‐resources settings. CAB+RPV LA also reduced the proportion with high‐level viraemia (>1000 copies/ml), a key factor in transmission risk. Most participants preferred injectable therapy, citing privacy and reduced stigma. The trial supports CAB+RPV LA as a viable option for those with adherence challenges, with potential public health benefits in Africa. On the other hand, real‐world data from the US‐based OPERA cohort evaluated CAB+RPV LA in treatment‐experienced people with HIV starting therapy with detectable viraemia. Of 3304 individuals, 368 began CAB+RPV LA while viraemic. Median HIV‐1 RNA at switch was low (120 copies/ml; IQR 61–2535). After a median follow‐up of 12 months, 88% achieved virological suppression, and only 1% experienced confirmed virologic failure [4]. Together, the results from the OPERA cohort and IMPALA support CAB+RPV LA for people struggling with adherence or tolerability on oral therapy. Beyond clinical efficacy, the implementation of this strategy, particularly in sub‐Saharan Africa, will depend on programmatic feasibility, cost and access. Its larger implementation in all settings could lead to a population benefit, reducing transmission events. The OPERA study and the IMPALA trial expand on earlier single‐centre real‐world data, including results from a San Francisco cohort [5]. Moreover, IMPALA results complement CARES trial data [6], suggesting a larger scale use of CAB+RPV LA might be of interest in resource‐limited settings. Notably, both CARES and IMPALA excluded participants with active hepatitis B virus (HBV) or with serological profiles associated with a high risk of reactivation (HBc antibody positive and HBs antibody <10 IU/l). In IMPALA, despite low HBV vaccination coverage (7% of participants), only one incident HBV infection was observed in the LA arm and was successfully managed with tenofovir‐containing ART. No reactivations were observed among the 86 participants with evidence of prior HBV and immunity (HBc antibody positive and HBs antibody >10 IU/l) [7]. These findings suggest that HBV reactivations were uncommon in this selected population, but they also highlight the importance of improving HBV vaccination coverage as these strategies are scaled up.

The MODERATO trial presented the first randomized data from West and Central Africa on two‐drug antiretroviral maintenance therapy [8]. Among adults with sustained virologic suppression, two‐drug therapy with DTG plus lamivudine (DTG‐3TC) or atazanavir/ritonavir‐lamivudine (ATV/r‐3TC) was non‐inferior to triple therapy at 96 weeks. Virologic failure rates and safety profiles were similar, supporting two‐drug therapy as a switch option in sub‐Saharan Africa. Two‐drug antiretroviral regimens, such as DTG‐3TC and injectable CAB+RPV LA, have been widely recommended by international guidelines from high‐income countries for ART simplification in virologically suppressed individuals over the last years [9, 10]. For the first time, these strategies have now also been recommended by WHO [11]. These two updates, covering both two‐drug therapy with DTG‐3TC and LA injectable CAB+RPV, supported by MODERATO [8], CARES [6] and IMPALA [3] trials, are particularly important, as they allow for the broader implementation of strategies already well established in high‐income countries to some point reducing the gap in the recommendations in high‐ and low‐income settings, while taking into account HBV co‐infection and cost‐related constraints.

Regarding novel agents, LA injectable drugs have been a major advance, but they may not be preferred, acceptable or feasible for all people with HIV or at risk, particularly given the cost and infrastructure required to sustain intramuscular delivery. For this reason, LA oral drugs are also needed. A phase 2b trial evaluated once‐weekly islatravir (ISL) plus ulonivirine (ULO) versus daily BIC/FTC/TAF in virologically suppressed adults. The study was discontinued early due to ISL‐associated reductions in lymphocyte and CD4+ counts, but virological suppression was maintained in all arms at 24 weeks [12]. A lower dose (2 mg) will be used in the coming trials involving ISL+ULO. ISL+ULO could potentially become the second weekly oral regimen, following ISL+lenacapavir, which is slightly ahead in development [13].

Another promising agent is MK‐8527, a once‐monthly oral drug on development for PrEP. In a phase 2a study, MK‐8527 was well tolerated, with no significant changes in lymphocyte or CD4+ counts and no HIV acquisitions during the study [14]. Although currently developed for prevention, MK‐8527 also highlights the potential of LA oral agents for future treatment strategies.

In 2025, USAID, the principal implementer of the US PEPFAR programme, has been radically reduced in scope and funding [2]. Modelling studies, including data presented at IAS 2025 and subsequently published, have suggested that these funding restrictions may lead to several additional millions of HIV acquisitions and deaths in the coming years, with a disproportionate impact on key populations [15, 16, 17]. In the face of these funding cuts, data on intermittent ART presented at the conference took on renewed relevance as a potential cost‐saving strategy. A systematic review and meta‐analysis compared intermittent ART dosing (3–5 days per week) to standard daily therapy, focusing on virologically suppressed, non‐pregnant adults without prior treatment failure or HBV. Across seven randomized trials with more than 1300 participants, most of them from high‐income settings, virological efficacy at 48 weeks was equivalent: 3% in both groups had HIV RNA >50 copies/ml. No significant differences were found in adherence, inflammation or drug resistance. However, emergent resistance was seen, mainly with regimens with a lower genetic barrier, underscoring the importance of regimen selection. Intermittent regimens were highly acceptable, improving treatment satisfaction and quality of life. The authors concluded that intermittent ART may be a feasible short‐term strategy during drug supply crises, based on comparable virological efficacy observed in selected, virologically suppressed populations with excellent adherence and no prior treatment failure. However, most studies in this meta‐analysis included highly selected, adherent populations and were largely based on NNRTI regimens, limiting generalizability to routine clinical practice. In addition, this approach is likely to be less forgiving in individuals with baseline adherence challenges, and its safety depends on the dosing strategy and the underlying regimen, with potentially higher risk when using lower–genetic‐barrier regimens [18]. Results in adolescent populations were less favourable. The BREATHER Plus study, conducted in several African countries, assessed short‐cycle therapy (5 days on/2 days off) versus continuous therapy in virologically suppressed adolescents on TLD. Among 470 participants, short‐cycle therapy was inferior: 10% experienced viral rebound compared to 5% in the continuous group, despite high adherence, with 96% self‐reported adherence and 92% adherence measured by MEMS caps in both arms. All nine participants who switched from short‐cycle to continuous therapy for confirmed viral rebound resuppressed, as did 10 of 14 participants who remained on short‐cycle therapy and 8 of 11 participants in the continuous therapy arm. Resistance was rare. The study highlighted that standard viral load monitoring (every 6–12 months) may be insufficient to safely support short‐cycle therapy in adolescents, emphasizing the need for tailored strategies in this group and in resource‐limited settings [19]. Considering these results as a whole, the appeal of intermittent ART is tempered by its potential risks. In resource‐limited settings, where such strategies might have a greater impact, a thorough evaluation is needed before recommending or implementing intermittent ART. It may be best reserved for subgroups with a proven history of excellent adherence over extended periods, and only with high genetic barrier combinations such as TLD. It remains to be determined whether switching to intermittent TLD offers greater economic and virological benefits compared to daily DTG‐based regimens in this setting.

### 2.2 Co‐infections and comorbidities

Significant findings were reported in the management of co‐infections, particularly mpox and tuberculosis (TB), as well as HBV risk with new ART regimens.

The UNITY trial, a phase III, multi‐country, double‐blind, placebo‐controlled study and the only randomized Clade II mpox trial to complete enrolment, evaluated tecovirimat, with most participants recruited in Brazil. Tecovirimat was well‐tolerated but did not significantly accelerate lesion resolution compared to placebo [20]. These results, consistent with other previous trials, highlight the ongoing need for effective mpox treatments, especially for those at high risk of severe outcomes. As was observed during the COVID‐19 pandemic, when therapeutic interventions were sometimes adopted beyond the populations studied in randomized trials despite limited validation [21], the UNITY trial underscores the importance of critically evaluating the use of tecovirimat in individuals with HIV and mpox. While some subgroups may still benefit from tecovirimat, for example, in UNITY, individuals with more severe disease or at higher risk of complications were treated outside the randomized comparison, current data do not support the use of tecovirimat for the treatment of mild to moderate mpox. Unfortunately, the list of other potentially useful drugs for the treatment of mpox is very limited, which underscores the importance of vaccination coverage. However, those most at risk of severe mpox, such as severely immunocompromised individuals with HIV, are also the ones in whom vaccine responses tend to be poorer.

A common clinical question in HIV medicine is whether the rapid initiation of ART may be harmful in individuals with suspected TB. Clinicians may choose to rule out active TB before starting ART. In high TB‐burden settings, the ATLAS trial evaluated the impact of empiric anti‐TB treatment and rifampicin dosing strategies in adults with HIV presenting with sepsis. While overall 28‐day mortality did not differ significantly in any arm, among those with confirmed TB, immediate therapy with conventional‐dose improved mortality [22]. Thus, early empiric TB treatment appears safe and may improve outcomes. The SaDAPT trial addressed whether same‐day ART initiation is safe for people with HIV presenting with presumptive TB. Same‐day ART was non‐inferior to waiting for TB diagnostic results in terms of viral suppression, retention in care and adverse events, with low and similar rates of immune reconstitution inflammatory syndrome (2% in each arm) [23]. Taken together, the findings from the ATLAS and SaDAPT trials suggest that in cases of high clinical suspicion of TB, particularly in severely ill individuals, empiric TB treatment combined with immediate initiation of ART might be an appropriate approach [22, 23].

Since the results of the ADVANCE trial were presented [24], weight gain and its association with ART have remained a significant topic in each HIV conference. At IAS 2025, data from the largest observational study to date on weight changes among virally suppressed people with HIV in the United States were presented. Of over 10,000 individuals, 12% experienced significant weight gain (≥10% with a body mass index [BMI] category shift), while 41% lost or maintained weight. Key predictors of weight gain included younger age, female sex, Black race, normal baseline BMI and lower baseline CD4 count. ART class, including integrase inhibitors and tenofofovir alafenanide (TAF), was not significantly associated with weight gain [25]. These findings tend to reinforce that socio‐demographic and clinical factors, rather than ART, drive the most clinically relevant weight gain, emphasizing the need for targeted, non‐ART‐focused interventions. Consistent with this, the DO‐IT trial showed that switching ART after obesity is established does not meaningfully reverse weight gain [25]. The ACTG A5391 “DO‐IT” trial evaluated whether switching from integrase inhibitor plus tenofovir alafenamide and emtricitabine (TAF/FTC) to doravirine‐based regimens would affect weight in people with HIV and obesity. The study enrolled the population most affected by weight gain (49% female; 53% Black; median BMI 39). Weight changes at 48 weeks were modest and not significantly different between arms, with all groups experiencing a slight reduction in weight and no differences between integrase strand transfer inhibitor (InSTI)‐ and doravirine‐based regimens, with or without TAF [26]. These findings indicate that switching ART does not meaningfully impact weight. While weight gain may not be a major concern for people with HIV in certain settings, such as young Caucasian men who have sex with men, it is more relevant in contexts where women and individuals of Black race predominate. Preventing significant weight gain is crucial, as modifying ART has shown limited or no benefit once obesity is established. Guidelines should emphasize the importance of prevention and lifestyle interventions whenever possible.

### 2.3 Special populations

Two communications focused on the paediatric population and pregnant women exposed to new antiretrovirals deserve a mention. Using data from the ODYSSEY trial, predictors of virological failure in children starting first‐line ART across Africa, Thailand and Europe were identified. Key predictors included younger age, low BMI‐for‐age and lower CD4% at ART initiation. DTG‐based ART was protective, and risk declined with increasing weight [27]. These findings can help guide timely interventions to prevent treatment failure in paediatric populations. They also align with the recommendation of the second generation InSTI for everyone with HIV, from newborns to elderly people. The Antiretroviral Pregnancy Registry analysed outcomes among pregnancies with prenatal exposure to CAB, either as treatment or PrEP. As of July 2024, 42 CAB‐exposed pregnancies were reported, resulting in 43 outcomes: 35 live births, one stillbirth, three spontaneous abortions and four induced abortions. Among live births, one congenital defect was reported. Most exposures occurred before conception, and the majority involved injectable CAB. No safety signals were identified, but the sample size remains small [28]. LA antiretrovirals for PrEP, including CAB LA and lenacapavir LA, are recommended by WHO and are being scaled up. Continued data collection is essential to better assess their safety during pregnancy, given that safety data during pregnancy remain limited. The EXPrESSIVE‐10 clinical trial, which evaluates MK‐8527 for women and adolescent girls in sub‐Saharan Africa, is not expected to require compulsory contraception from participants, thereby enabling the eventual collection of safety data during pregnancy. This approach, already implemented in PURPOSE‐1 [29], is likely the most appropriate for scaling up the use of MK‐8527, if approved, for a population simultaneously at risk of HIV acquisition and pregnancy.

## 3 CONCLUSIONS

In summary, the conference illustrated how far HIV treatment has progressed, while also highlighting the remaining gaps between scientific innovation and real‐world implementation. Advances in LA and simplified ART regimens are expanding therapeutic options and have the potential to improve adherence, quality of life and programmatic efficiency, including resource‐limited settings. At the same time, the mixed results seen with intermittent therapy, the absence of effective treatment for mpox, and the persistent challenges of TB, weight gain and paediatric treatment underscore that important clinical needs remain unmet. Despite funding constraints being mentioned in most sessions, the tone of the conference remained consistently positive, focused on finding alternatives in challenging times and pushing forward despite adversity. The WHO's recommendation, for the first time, to use two‐drug ART oral and injectable combinations in resource‐limited settings is a clear testament to that spirit.

The slogan of the conference was ***We Will Not Go Back***, and all stakeholders should work to uphold and support this message, advancing the achievements in the HIV field despite the difficult global context. While innovation gaps across regions will persist, efforts must continue to minimize them.

## COMPETING INTERESTS

Ezequiel Cordova has received personal fees from and participated in advisory boards for ViiV, Janssen and MSD; has received funding for research from ViiV, all outside the current work. Simiso Sokhela's institution has received research funding from Janssen, MSD, ABBVIE and Gilead, and personal honoraria for participating in an ABBVIE advisory board. Jasmini Alagaratnam has received research grants from Gilead Sciences, National Institute for Health and Care Research (NIHR), Imperial Biomedical Research Centre (BRC) and British HIV Association (BHIVA), paid to her institution, and has received speaker's fees and/or advisory board honoraria and/or conference travel support from Gilead Sciences Ltd, MSD and ViiV Healthcare, unrelated to this work. Juan Ambrosioni has received personal fees from and participated in advisory boards for ViiV, Gilead, Janssen and MSD; has received funding for research and/or educational events from ViiV, Gilead and MSD; and has been a member of data safety monitoring boards for HIPRA and Grifols, all outside the current work.

## AUTHORS’ CONTRIBUTIONS

The authors served as Rapporteurs for Clinical Science (Track B) at the IAS 2025 conference. The presentations discussed were selected by the lead rapporteur (JA). EC, SS and JAl prepared initial drafts based on the rapporteur sessions. JA compiled all reports and harmonized the content. The draft underwent multiple rounds of review by all co‐authors, and the final manuscript reflects their collective contributions and opinions.

## FUNDING

The authors acted as Rapporteurs and worked on the manuscript on a voluntary basis. The authors received support to attend IAS 2025.

## Data Availability

Data sharing is not applicable to this article as no datasets were generated or analysed during the current study.
